# Supplementary material for: Salivary Interleukin Levels in Oral Squamous Cell Carcinoma and Oral Epithelial Dysplasia: Findings from a Sri Lankan Study
Source: Cancers (Basel). 2023 Feb 28;15(5):1510. doi: 10.3390/cancers15051510 (PMC10001283; doi:10.3390/cancers15051510)
Supplement: Supplementary file 1 [file cancers-15-01510-s001.zip › Supplementary Data S1.pdf]

Supplimentary Data S1: Raw data values for salivary biomarkers in the study participants

Age	Sex	Group	IL1B (pg/ml)	IL6(pg/ml)	IL8 (pg/ml)
70	male	Control	18.38	1.61	0
49	male	Control	4.72	12.4	0
78	male	Control	12.04	16.53	93.55
36	male	Control	23.04	3.27	0
68	male	Control	7.99	10.73	0
71	male	Control	13.17	92.43	88.7
66	female	Control	30.95	26.81	62.12
71	male	Control	3.36	4.09	0
64	male	Control	15.55	0	0
65	female	Control	2.78	20.7	0
69	male	Control	0.38	6.69	0
47	male	Control	0	6.92	0
48	male	Control	0	0	292.98
43	male	Control	1.69	26.45	0
57	male	Control	80.16	0	0
69	female	Control	15.46	23.08	120.58
70	male	Control	16.08	1.28	0
59	male	Control	109.27	15.62	9.2
57	male	Control	1.93	5.26	6.24
61	male	Control	NT	14.81	240.26
54	female	Control	NT	21.89	100.51
63	female	Control	NT	21.82	444.76
65	male	Control	NT	535.88	0
69	male	Control	NT	0	343.82
59	male	Control	5.38	3.71	0
69	male	Control	10.61	1.88	0
72	female	Control	6.82	1.84	0
55	male	Control	5.72	6.92	67.56
63	male	Control	1.25	1.37	0
79	male	Control	20.01	81.49	328.29
51	male	OSCC	NT	7.78	NT
53	male	OSCC	35.58	6.08	3592.99
56	male	OSCC	84.85	55.45	3637.81

70	male	OSCC	NT	1127.75	2031.66
75	female	OSCC	NT	2243.48	NT
67	male	OSCC	0	3.48	0
67	male	OSCC	78.35	6473.53	6473.53
64	male	OSCC	312.59	1237.97	5707.18
56	male	OSCC	148.03	402.33	4805.16
69	female	OSCC	NT	1588.57	3138.29
52	male	OSCC	4.94	99.47	3221.61
68	male	OSCC	NT	260.55	907.32
63	male	OSCC	NT	6047.34	4956.42
58	male	OSCC	NT	1933.34	NT
67	male	OSCC	35.93	354.92	1389.03
42	female	OSCC	0.96	12.63	601.83
55	male	OSCC	20.96	1456.31	2112
51	male	OSCC	9.52	288.57	1204.17
43	male	OSCC	111.03	1065.15	2644.85
33	female	OSCC	9.45	607.31	2856.18
66	male	OSCC	48.75	10.69	3975.73
73	male	OSCC	0	2731.28	5902.5
72	male	OSCC	NT	7.54	2219.35
61	male	OSCC	13.72	495.59	3492.23
76	male	OSCC	NT	175.55	NT
78	female	OSCC	NT	1942.99	NT
41	male	OSCC	176.6	950.15	5604.1
65	male	OSCC	146.47	1140.94	5075.13
70	male	OSCC	25.36	3377.53	5157.18
42	male	OSCC	5.64	0	1113.1
76	male	OSCC	145.99	4245.55	4245.55
47	male	OSCC	53.82	538.41	2821.66
73	male	OSCC	4.32	0	2633.22
58	male	OSCC	188.56	1462.14	4945.06
52	female	OSCC	NT	552.81	4997.76
56	male	OSCC	25.84	529.05	3086.24
55	male	OSCC	NT	146.17	3572.19
56	female	OED	15.05	6.16	0
61	male	OED	134.51	582.63	1816.28
30	male	OED	3.39	3.76	0
36	male	OED	25.59	12.65	12.59

58	male	OED	0	1.44	0
72	male	OED	7.52	41.94	15.03
41	female	OED	6.16	0	378.2
66	male	OED	NT	7.57	326.71
40	male	OED	NT	66.66	478.1
53	female	OED	NT	58.83	528.98
54	male	OED	NT	0	409.32
58	female	OED	NT	47.28	254.22
60	male	OED	NT	91.95	226.46
70	male	OED	3.5	110.71	980.12
60	male	OED	11.71	11.96	25.49
69	male	OED	2.06	0	0
29	male	OED	2.82	5.91	0
60	male	OED	73.93	17.69	19.95
80	male	OED	16.73	6.53	0
66	male	OED	0	6.83	1.35
55	male	OED	179.16	163.75	303.87
46	female	OED	3.13	176.01	452.07
67	male	OED	0	87.29	86.93
57	male	OED	3.39	0.96	0
55	male	OED	3.5	0.74	0
52	male	OED	35.8	12.59	0
72	male	OED	27.93	29.05	0
59	female	OED	31.3	249.29	337.6
45	male	OED	3.6	16.15	0
59	male	OED	22.41	21.57	3.77

NT= Data not obtained, 0 = Indicates samples that had an absorbence reading equivelant to or lower than the absorbence value of the Blank.
