# Supplementary material for: Salivary Interleukin Levels in Oral Squamous Cell Carcinoma and Oral Epithelial Dysplasia: Findings from a Sri Lankan Study
Source: Cancers (Basel). 2023 Feb 28;15(5):1510. doi: 10.3390/cancers15051510 (PMC10001283; doi:10.3390/cancers15051510)
Supplement: Supplementary file 1 [file cancers-15-01510-s001.zip › Supplementary Data S2.pdf]

Supplementary Data S2: Associations between salivary IL1β, IL6 and IL8 and risk factors in study groups

Results Summary

The associations between salivary biomarkers and smoking, alcohol, betel quid chewing, family history of cancer, mouthwash use, and co-morbidities were assessed; the results are demonstrated in Figures 1-6. We could not detect any statistically significant associations or consistent patterns between smoking, alcohol, betel quid and family history of cancer with the studied biomarkers (Supplemental Figure 1-4). Regarding the mouthwash use, in the OSCC group, mouthwash users had higher biomarker levels while in the OED group, mouthwash users had a lower biomarker level (Supplemental Figure 5). Concerning the salivary biomarker levels of patients with co-morbidities, there was a pattern where the patients with co-morbidities have a higher biomarker level compared to those who did not had any co-morbidity. The IL6 level of the co-morbid group was significantly higher (p=0.014) compared to the patients without any co-morbidity in the OED group. Unequal and small sample sizes in risk factor sub-categories were limitation of this analysis.

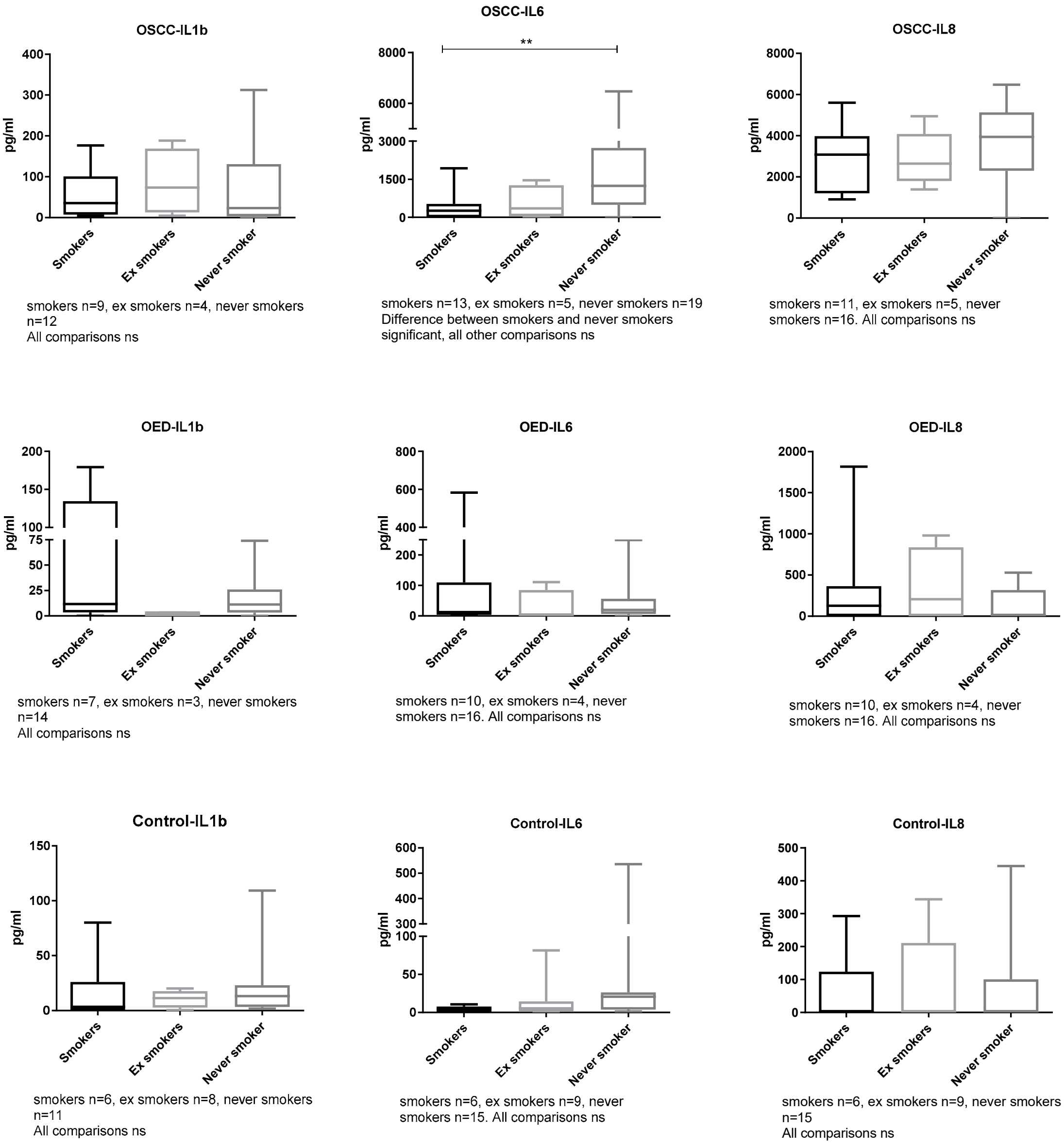

Supplemental Figure S1: Relationships between salivary biomarkers and smoking
Box and Whisker plots, box indicate horizontal bars for median and inter-quartile range, whiskers indicate minimum and maximum values, Statistical tests were Kruskal Wallis and Dunn's test, p values < 0.05 are indicated by *, p values <0.01 are indicated by **, ns: statistically non-significant p>0.05. **This figure indicates that IL6 level in never smokers was significantly high compared to smokers in the OSCC group. All other comparisons were statistically non-significant. There was no clear pattern of salivary biomarkers and smoking subcategories in the study groups.**

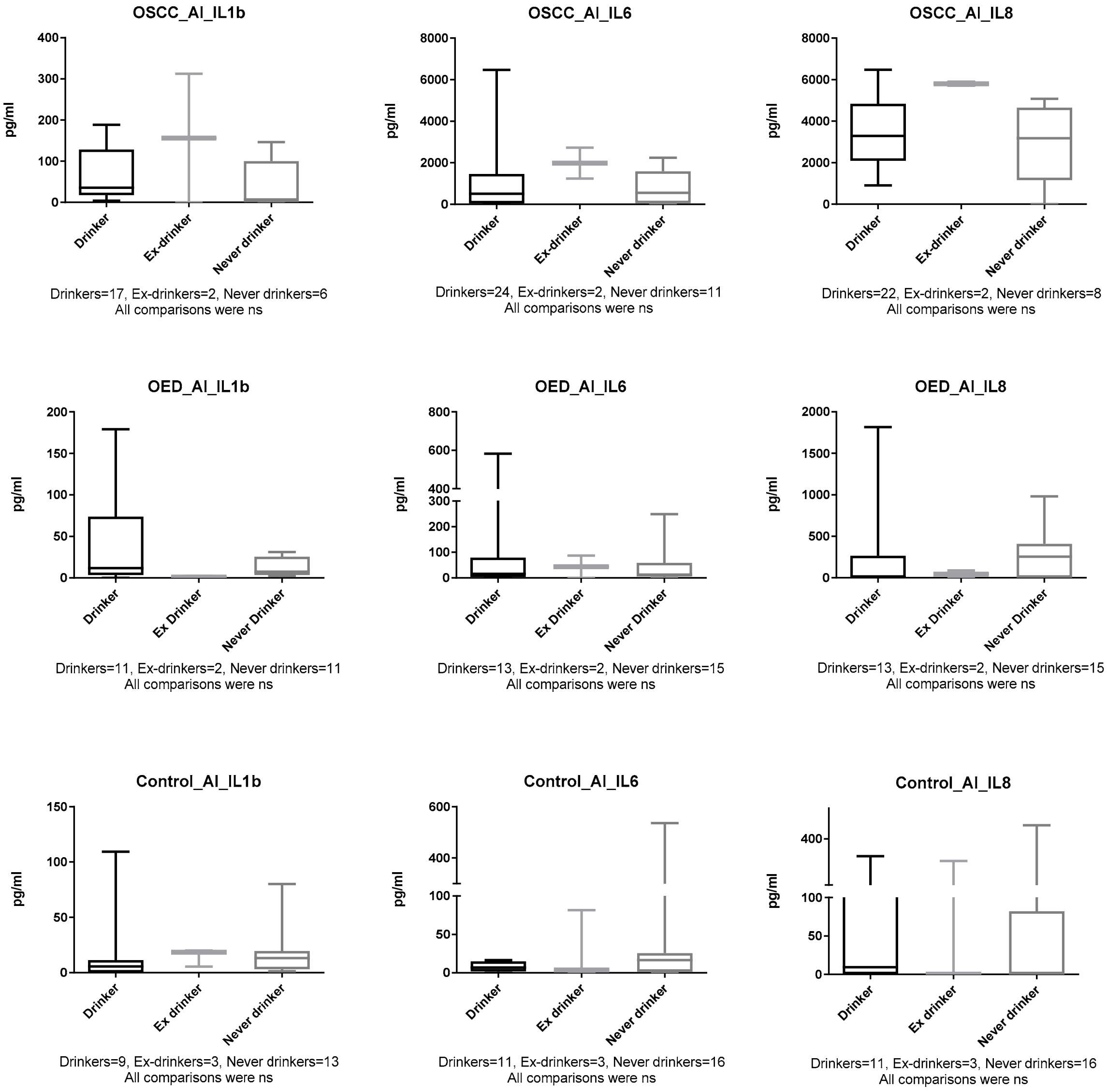

Supplemental Figure S2: Relationships between salivary biomarkers and alcohol
Box and Whisker plots, box indicate horizontal bars for median and inter-quartile range, whiskers indicate minimum and maximum values, statistical tests were Kruskal Wallis and Dunn's test, ns: statistically non-significant p>0.05. **There were no significant relationships or clear pattern between salivary biomarkers and alcohol habit**

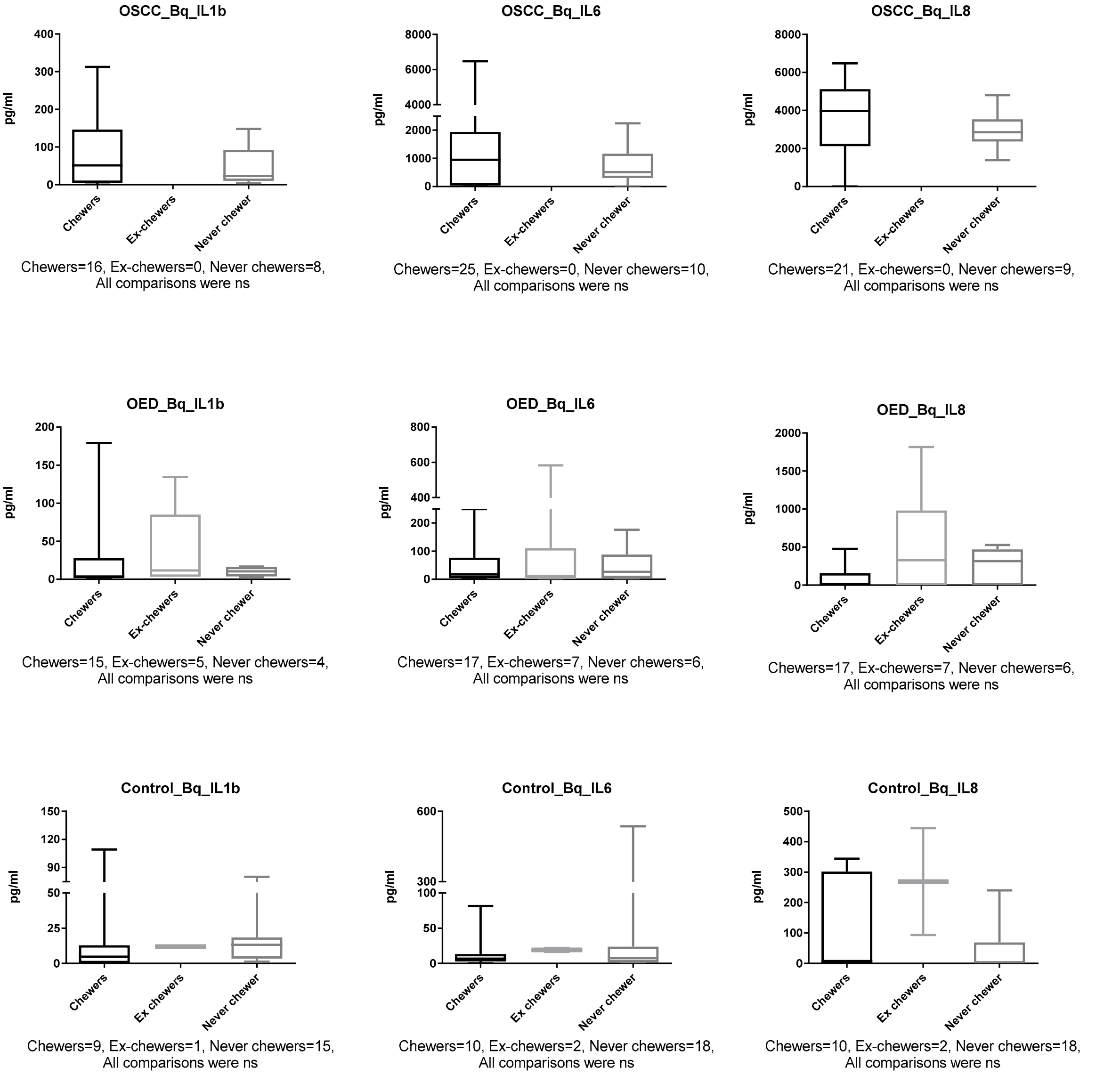

Supplemental Figure S3: Relationships between salivary biomarkers and betel quid
Box and Whisker plots, box indicate horizontal bars for median and inter-quartile range, whiskers indicate minimum and maximum values, statistical tests were Mann Whitney U, Kruskal Wallis and Dunn's tests, ns: statistically non-significant p>0.05. **This figure revealed that betel quid chewers in the OSCC group had high biomarker values compared to non-chewers, without statistical significance.**

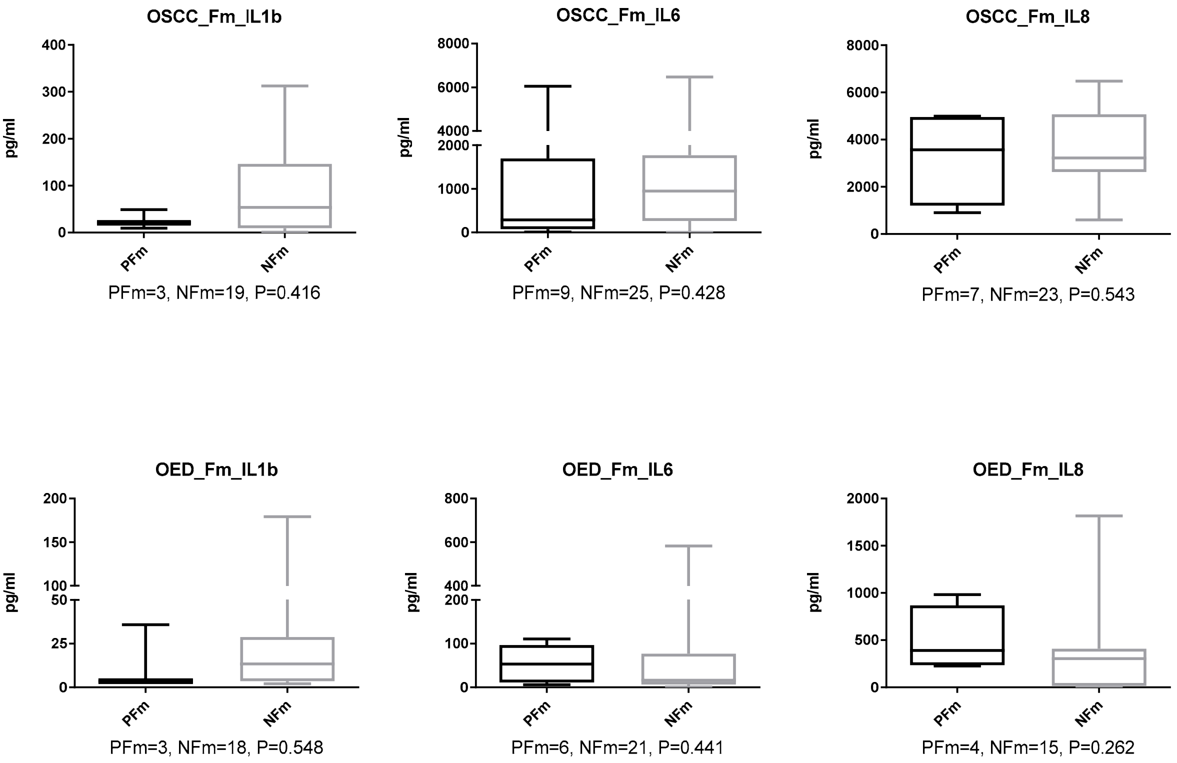

Supplemental Figure S4: Relationships between salivary biomarkers and family history of cancer
Box and Whisker plots, box indicate horizontal bars for median and inter-quartile range, whiskers indicate minimum and maximum values, statistical test was Mann Whiteney U test, ns: statistically non-significant p>0.05. **There were no statistically significant differences.**

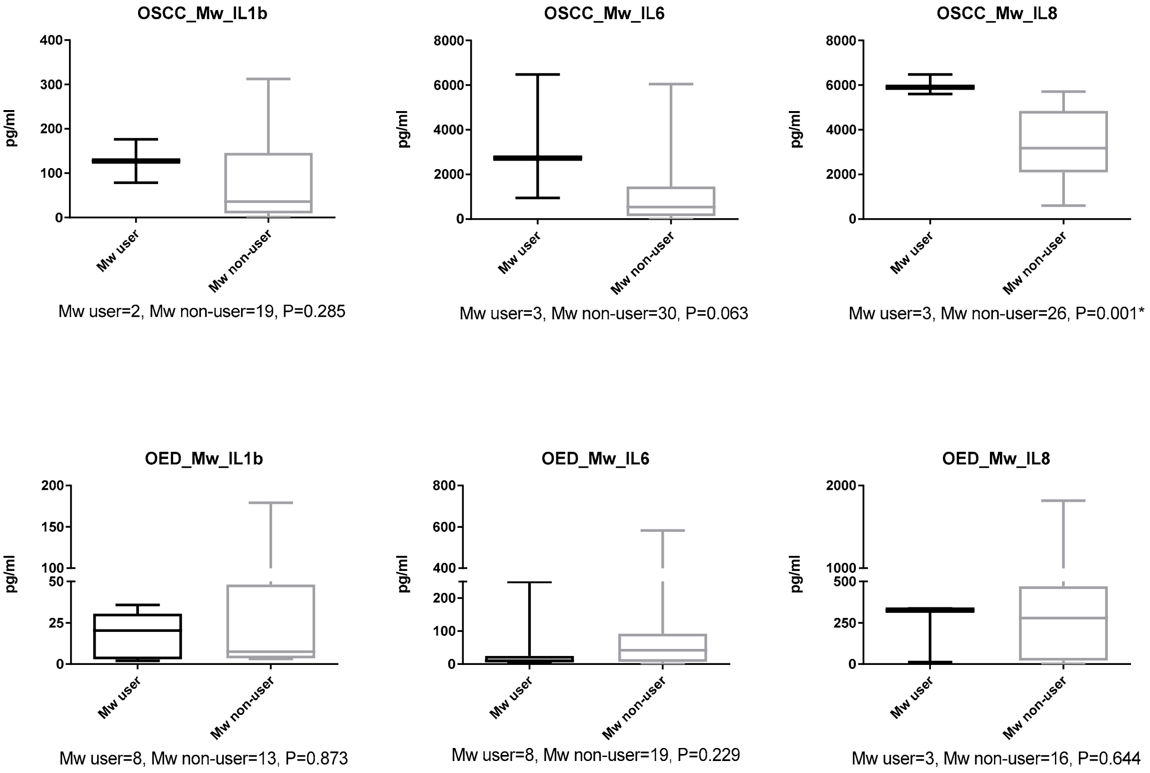

Supplemental Figure S5: Relationships between salivary biomarkers and mouthwash use. Box and Whisker plots, box indicate horizontal bars for median and inter-quartile range, whiskers indicate minimum and maximum values, statistical tests were Mann Whitney U tests, ns: statistically non-significant p>0.05. **In the OSCC group, MW users had higher biomarker levels while in the OED group, MW users had a lower biomarker level.**

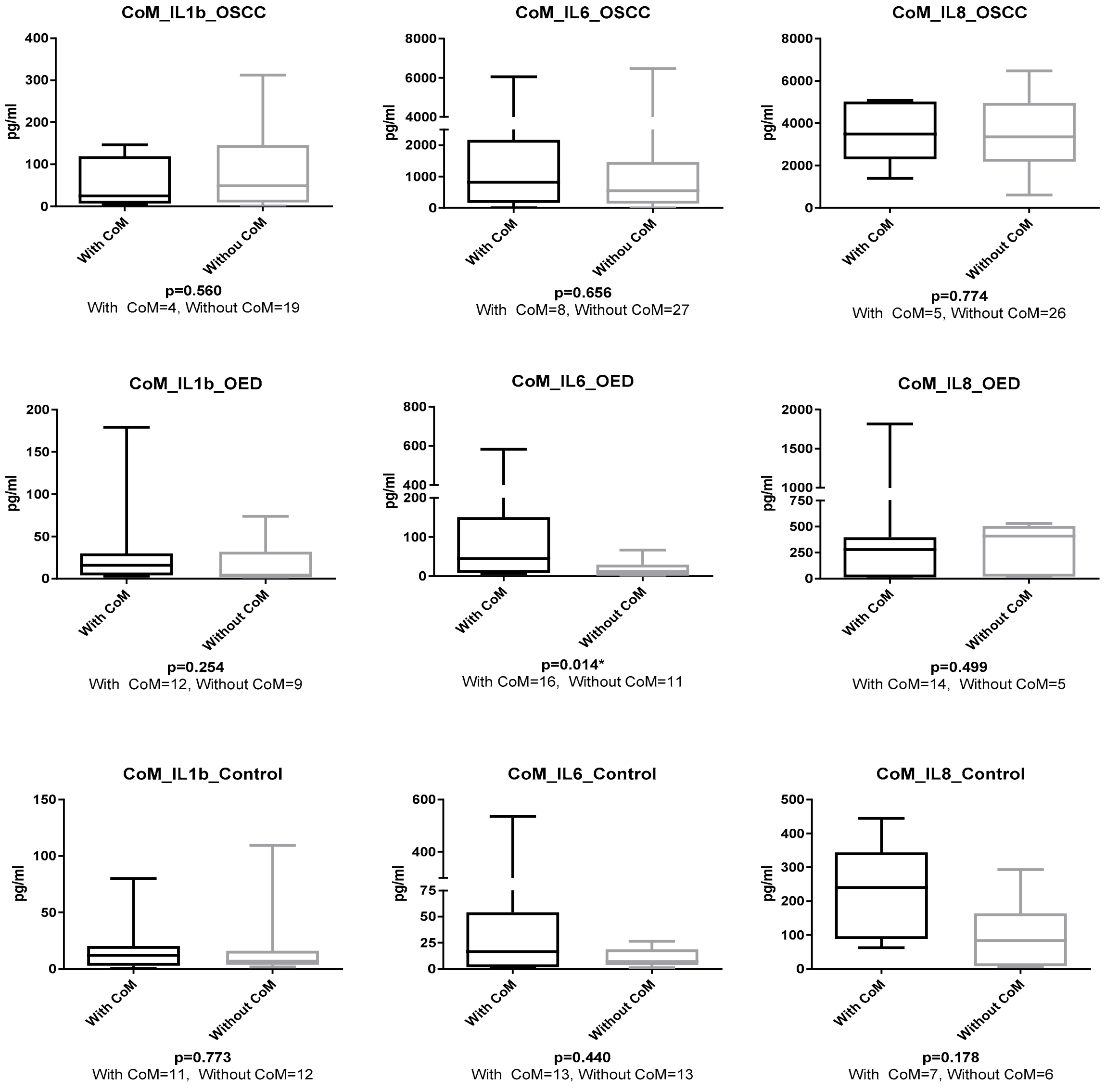

Figure S6: Relationships between salivary biomarkers and co-morbidities. Box and Whisker plots, box indicate horizontal bars for median and inter-quartile range, whiskers indicate minimum and maximum values, statistical test was Mann Whitney U test, *statistically significant $p<0.05$. **Group with co-morbidities had a higher biomarker level, the IL6 level of the co-morbid group was significantly higher compared to the patients without any co-morbidity in the OED group.**
